# NoiseNet, a fully automatic noise assessment tool that can identify non-diagnostic CCTA examinations

**DOI:** 10.1007/s10554-024-03130-x

**Published:** 2024-05-15

**Authors:** Emma Palmquist, Jennifer Alvén, Michael Kercsik, Måns Larsson, Niklas Lundqvist, Ola Hjelmgren, Erika Fagman

**Affiliations:** 1 Department of Radiology, Institute of Clinical Sciences, University of Gothenburg, Gothenburg, Sweden; 2 Department of Radiology, Sahlgrenska University Hospital, Region Västra Götaland, Gothenburg, SE-413 4 Sweden; 3 Computer Vision, Department of Electrical Engineering, Chalmers University of Technology, Gothenburg, Sweden; 4 Department of Radiology, Alingsås Hospital, Region Västra Götaland, Alingsås, Sweden; 5 Eigenvision AB, Gothenburg, Sweden; 6 Department of Molecular and Clinical Medicine, Institute of Medicine, University of Gothenburg, Gothenburg, Sweden; 7 Pediatric Heart Centre, Queen Silvias Pediatric Hospital, Sahlgrenska University Hospital, Gothenburg, Sweden

**Keywords:** Coronary computed tomography angiography, Noise, Signal to noise, Image quality, Deep learning, Artificial intelligence

## Abstract

Image noise and vascular attenuation are important factors affecting image quality and diagnostic accuracy of coronary computed tomography angiography (CCTA). The aim of this study was to develop an algorithm that automatically performs noise and attenuation measurements in CCTA and to evaluate the ability of the algorithm to identify non-diagnostic examinations. The algorithm, “NoiseNet”, was trained and tested on 244 CCTA studies from the Swedish CArdioPulmonary BioImage Study. The model is a 3D U-Net that automatically segments the aortic root and measures attenuation (Hounsfield Units, HU), noise (standard deviation of HU, HUsd) and signal-to-noise ratio (SNR, HU/HUsd) in the aortic lumen, close to the left coronary ostium. NoiseNet was then applied to 529 CCTA studies previously categorized into three subgroups: fully diagnostic, diagnostic with excluded parts and non-diagnostic. There was excellent correlation between NoiseNet and manual measurements of noise (*r* = 0.948; *p* < 0.001) and SNR (*r* = 0.948; <0.001). There was a significant difference in noise levels between the image quality subgroups: fully diagnostic 33.1 (29.8–37.9); diagnostic with excluded parts 36.1 (31.5–40.3) and non-diagnostic 42.1 (35.2–47.7; *p* < 0.001). Corresponding values for SNR were 16.1 (14.0–18.0); 14.0 (12.4–16.2) and 11.1 (9.6–14.0; *p* < 0.001). ROC analysis for prediction of a non-diagnostic study showed an AUC for noise of 0.73 (CI 0.64–0.83) and for SNR of 0.80 (CI 0.71–0.89). In conclusion, NoiseNet can perform noise and SNR measurements with high accuracy. Noise and SNR impact image quality and automatic measurements may be used to identify CCTA studies with low image quality.

## Introduction

Coronary computed tomography angiography (CCTA) is a non-invasive method for the detection and quantification of coronary artery disease and is one of the recommended first line imaging modalities in the work-up of patients with suspicion of chronic coronary syndrome [1]. For accurate detection of coronary lesions, high image quality is crucial and poor image quality has been shown to significantly increase the number of false positive CCTA examinations [2]. CCTA is a technically challenging procedure, and the final image quality is determined by both patient-related and technical factors [3]. The main determinants of image quality are contrast attenuation of the coronary arteries, coronary motion, and image noise level [3]. The noise level of an examination depends on the number of photons reaching the detector which is influenced by several technical and patient related factors. Body Mass Index and the area of solid tissue at the level of the aortic root are examples of patient related factors that have been previously shown to affect the noise level and image quality of CCTA [2, 4, 5].

Quantitative analysis of noise can be performed by placing a region of interest (ROI) in a specific area and measure the mean attenuation (measured in Hounsfield units, HU) representing the signal, and the standard deviation of the HU value representing the image noise. By these two measurements the signal-to-noise ratio (SNR), defined as the quotient between mean attenuation and image noise, can be calculated [3, 6]. In several previous studies, noise measurement in CCTA has been performed in the aortic root at the level of the ostium of the left main coronary artery [4, 7, 8]. Image noise at this level has been shown to influence image quality of CCTA [4]. Since both the level of contrast enhancement and the noise level influence image quality, signal-to-noise ratio can be used as another measurement of image quality of CCTA [8, 9].

High image quality in CCTA is important not only for a correct diagnosis in the clinical setting but also for the validity of data in a research setting. The Swedish CArdioPulmonary BioImage Study (SCAPIS) is a Swedish population-based prospective study where 27 385 participants aged 50–64 years underwent CCTA [10]. In SCAPIS, a total of 1805 participants (6.6%) had poor image quality in proximal coronary segments [11]. The evaluation of image quality was made by visual assessment by the reader and is likely to be variable. Hence, objective measurements of image quality might be of value in further evaluation of large CCTA studies, like SCAPIS.

The aim of this study was to develop an algorithm that can automatically perform attenuation and noise measurements in CCTA. We hypothesize that (i) an automatic algorithm can perform attenuation and noise measurements on par with a radiologist and (ii) automatic attenuation and noise measurements can be used to evaluate image quality and detect non-diagnostic examinations.

## Materials and methods

### CCTA datasets

The automatic aortic root segmentation algorithm, named “NoiseNet”, was trained, validated and tested on CCTA studies from SCAPIS [10]. The studies used came from a cohort of 529 previously analyzed CCTA studies originally used for plaque segmentation, where all cases contained at least one coronary plaque [12]. The studies were categorized into three image quality subgroups based on a previously performed analysis of the coronary artery tree [12]: fully diagnostic (*n* = 159); diagnostic with excluded parts (*n* = 341) and non-diagnostic (*n* = 29).

Out of a total of 529 cases, 29 had overall low image quality and were not used for NoiseNet model training. From the remaining 500 cases, we randomly selected 147 cases for training and 37 cases for validation. A separate testset of 60 cases, for testing the performance of NoiseNet in automatic noise measurement, was randomly selected and not used in training or validation. To evaluate the influence of noise level on image quality, the algorithm was applied to all 529 CCTA studies.

The selection and plaque analysis procedure of the CCTA studies has been described in detail in a previous publication [12]. The analysis procedure included all coronary arteries down to a diameter of 2 mm and was performed with a semi-automatic software with vessel wall and lumen contouring and manual marking of all coronary plaques. The analysis was performed as a two-step process by four primary readers and one expert secondary reader (author EF). Parts of the coronary artery tree with impaired image quality, where lumen and vessel wall contours could not be accurately defined by visual assessment, were manually marked (start and end points) as excluded. The reason for exclusion was categorized by the reader as high noise level, motion artifacts or other. The total length of excluded and included parts of the coronary artery tree in each study was calculated. Cases where parts of the vessel tree were excluded were categorized as “diagnostic with excluded parts”. In cases where the whole study was considered non-diagnostic due to poor image quality, the whole CCTA examination was marked as excluded and categorized as “non-diagnostic”. Finally, cases where no exclusions were made were categorized as “fully diagnostic” [12].

The SCAPIS study was approved as a multi-center study by the ethical review board in Umeå (# 2010-228-31 M). The participants gave written informed consent. The current sub-study was approved by the ethical review board in Gothenburg, (#570 − 18).

### CCTA protocol

The CCTA protocol in SCAPIS has been previously described in detail [10]. Briefly, computed tomography (CT) scanning was performed using a Somatom Definition Flash scanner with a Stellar detector (Siemens Medical Solutions, Forchheim, Germany). Five different CCTA protocols were used depending on heart rate, heart rate variability, presence of calcifications and body weight. Iohexol (Omnipaque, GE Healthcare, 350 mg I/ml) was used as a contrast medium. A β-blocker (metoprolol) was administered to decrease the heart rate and sublingual glyceryl trinitrate to dilate the coronary arteries.

### Automatic aortic root segmentation – training and validation of NoiseNet

Our approach to automatic determination of noise levels in CT imaging requires segmentation of the zone where noise is measured. This segmentation was achieved using a convolutional neural network (CNN) based approach. A 3D UNet [13] was trained to segment the aortic root, the left main coronary artery (LM), and the proximal right coronary artery (RCA). The UNet takes the CCTA study resampled to a resolution of (0.9, 0.66, 0.66) mm per pixel as input.

For training and validation of the CNN, 184 CCTA studies were manually annotated by a single reader (MK, specialist in radiology with five years of experience). The aortic root was manually segmented using a DICOM viewer from The Research Consortium for Medical Image Analysis (RECOMIA) [14]. The ostium of the LM and RCA were manually marked. During training of the CNN, any labelled artery pixels that were far away from the aorta were ignored, and pixels right at the edge of the segmentations were also excluded as we consider these pixels as neither well-defined nor precise. We believe that these modifications helped to develop a robust CNN for aortic root segmentation without affecting the precision of the final noise measurements. The model was trained using cross entropy loss and the Nadam optimizer [15]. The UNet was modified by adding dropout layers with a dropout rate of 0.5 before the last and second to last convolution layers. In addition, L2 weight regularization with a factor of 10^− 3^ was applied during training. The learning rate was initially set to 10^− 4^ and was multiplied by a factor 0.5 if the validation loss had not decreased for five epochs. One training epoch was defined as 20,000 iterations and every 500 epochs, the model was run on the entire training set to enable higher sampling rate of the high loss areas. The training continued until the validation loss had not decreased for 10 epochs (which took 1500 epochs) and the model with the highest Dice coefficient on the validation set was chosen as the final model. During inference, the 3D UNet was applied to the entire CCTA study in a sliding window manner. To postprocess the results, all connected components except the largest were removed for each class, and all holes were filled.

**Fig. 1:**
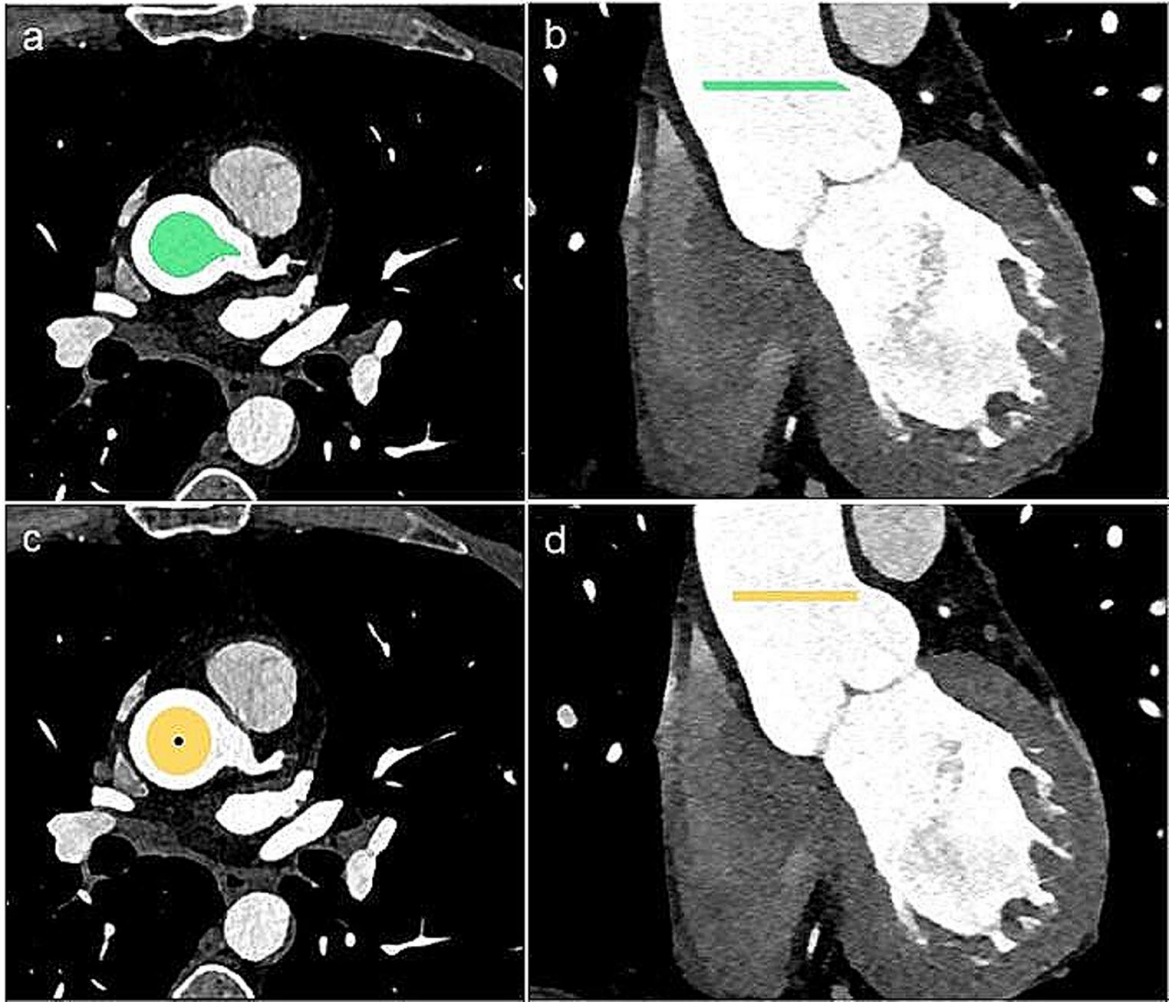
Automatic (**a**, **b**) and manual (**c**, **d**) segmentation for noise measurements in the aortic root. For automatic measurement, the aortic root was segmented by NoiseNet (**a**, **b**). The green volume in a and b was automatically generated with the center at the level of contact point of the left main coronary artery (LM). The volume consists of seven image slices with a slice thickness of 0.5 mm. For manual measurements, the reader placed a point (black dot, c) at the center of the aortic root at the level of the ostium of the LM. From this point, a region of interest (ROI) with a diameter of 15 mm was generated (yellow area, c). d shows a coronal reformat with the level of measurement marked with a yellow line

To determine the location for automatic noise measurement, the segmentations of the aortic root and the LM were used. By dilating the LM segmentation by one pixel in all directions in the axial plane, the pixel of the dilated LM segmentation that overlapped with the original aorta segmentation was defined as the contact point segmentation. The contact point of the LM was used to define the center axial slice of the zone for noise measurement and the zone was defined by adding three slices above and below the center slice. Next, the aortic root segmentation was shrunk by 1/3 of its radius in each axial slice. The resulting volume of segmented aortic root at the level of the LM was used for generating automatic noise measurements (Fig. 1a, b).

### Test of NoiseNet

The segmentation of the aortic root by NoiseNet was tested by calculating the Dice coefficient on the validation set (*n* = 37), since there were no manual annotations of the aortic root in the test set.

The final task for NoiseNet, to measure noise in the aortic root, was tested in the testset (*n* = 60), where NoiseNet was compared to manual measurements. The CCTA studies were manually analyzed using a DICOM viewer from RECOMIA. All manual measurements were performed by a single reader (EP, radiology resident with three years of experience). The reader manually placed a ROI with a diameter of 15 mm in the center of the aortic root at the level of LM (Fig. 1c, d). For each ROI, the mean attenuation and noise were calculated. From these measurements the signal-to-noise ratio, was calculated. Each of these variables were also automatically assessed by NoiseNet.

### Influence of noise on image quality

NoiseNet was applied to 529 CCTA studies generating attenuation and noise levels and signal-to-noise ratio in the aortic root. The output variables were compared to the available image quality data. The CCTA studies were divided into three subgroups based on the previously performed manual analysis where all parts of the coronary tree with impaired image quality were marked. The groups were: (1) Fully diagnostic; (2) Diagnostic with excluded parts; (3) Non-diagnostic. In group 2, the vessel lengths of included and excluded parts were used for calculating the proportion of the coronary artery tree that had been excluded.

### Statistical analysis

For statistical analysis SPSS version 28 (IBM corp.) was used. The data was tested for normal distribution using Kolmogorov-Smirnov-test. Quantitative variables are presented as mean ± standard deviation (SD) or median and interquartile range (IQR). Difference between groups was evaluated using two-tailed paired t-test or Mann Whitney U-test. Correlation and systematic error were assessed using Spearman correlation coefficient and Bland-Altman plots. The ability to predict a non-diagnostic study was estimated by using receiver operating characteristic (ROC) curve and calculating the area under curve (AUC). A p-value < 0.05 was considered statistically significant. Model segmentation performance was evaluated using the Dice coefficient.

**Table 1:** Characteristics of the study populations

	NoiseNet training and testing		Dataset for evaluation of the influence of noise on image quality			
	**Training/validation** **set**	**Test set**	**All studies**	**Fully diagnostic studies**	**Diagnostic studies with excluded parts**	**Non-diagnostic studies**
*Sample size*	184	60	528	158	341	29
*Age, years*	61.3 (56.9–63.5)	60.8 (58.8–63.3)	61.6 (58.7.-63.6)	61.4 (58.4–63.5)	61.6 (58.8–63.6)	62.0 (59.6–64.1)
*Female sex, n (%)*	34 (18.5)	15 (25.0)	82 (15.5)	30 (19.0)	49 (14.4)	3 (10.3)
*Body mass index, kg/m* ^*2*^	26.9 (24.6–29.3)	26.1 (23.5–28.5)	26.6 (24.3–29.0)	26.0 (23.6–27.9)	26.8 (24.7–29.1)	28.8 (25.5–32.2)
*Height, cm*	177.0 (171.0-183.0)	176.5 (170.0-180.0)	177.0 (171.0-182.0)	176.0 (170.0-181.0)	177 (171.0-182.0)	177 (171.5–182)
*Weight, kg*	84.3 (76.4–92.0)	79.5 (72.0–89.0)	82.7 (75.0-91.5)	80.0 (71.0–88.0)	83.9 (76.0-92.1)	93.0 (80.0-104.3)
*Waist circumference, cm*	98.0 (90.0-104.0)	95.0 (89.0-101.0)	98.0 (91.0-104.0)^a^	97.0 (87.5–102.0)	98.0 (92.0-105.0)	102.0 (94.0-110.5)
*Current smoker, n*	49 (27.7)^b^	19 (31.7)	166 (32.7)^c^	51 (32.3)	108 (31.7)	7 (24.1)
*Total cholesterol. mmol/L*	5.6 (5.0-6.3)	5.6 (4.8–6.4)	5.7 (5.1–6.4)^a^	5.8 (5.1–6.5)	5.7 (5.0-6.4)	5.7 (5.2–6.1)
*Systolic blood- pressure, mm Hg*	134.5 (123.0-152.0)	137.0 (124.5-153.5)	136.0 (124.0-151.8)	134.0 (122.0-146.3)	138 (124.0-152.0)	144.0 (135.5–161.0)
*SCORE*	3.1 (1.3–4.8)	3.1 (1.6–4.9)	3.4 (1.9–4.9)^a^	3.2 (1.7–4.9)	3.4 (2.0-4.9)	3.9 (2.6–4.8)
*CACS*	31.0 (5.0-120.5)^d^	18.0 (3.0-102.0)^a^	47.0 (7.0-168.5)^e^	41.0 (7.0-133.0)	47.5 (7.0-201.3)	96.0 (11.0-256.0)
*CCTA radiation dose, mSv*	1.4 (1.1–1.8)	1.4 (1.1–1.6)	1.4 (1.2–1.8)	1.4 (1.1–1.7)	1.5 (1.2–1.8)	1.7 (1.3–2.3)
*Glyceryltrinitrate administered, n (%)*	173 (94.0)	57 (95.0)	491 (93.0)	145 (91.8)	321 (94.1)	25 (86.2)

Values are in median (interquartile range) or n (%). ^a^One case is missing; ^b^7 cases are missing^; c^20 cases are missing; ^d^3 cases are missing; ^e^6 cases are missing. SCORE = Systematic Coronary Risk Evaluation, CACS = coronary artery calcium score

## Results

### Study populations

The characteristics of the study populations used for training and testing of NoiseNet and for evaluating the influence of noise on image quality are presented in Table 1.

### Test of NoiseNet segmentation

For the 37 studies in the validation set the mean Dice coefficient was 0.96 for the aortic root segmentation.

### Test of NoiseNet measurements

**Fig. 2:**
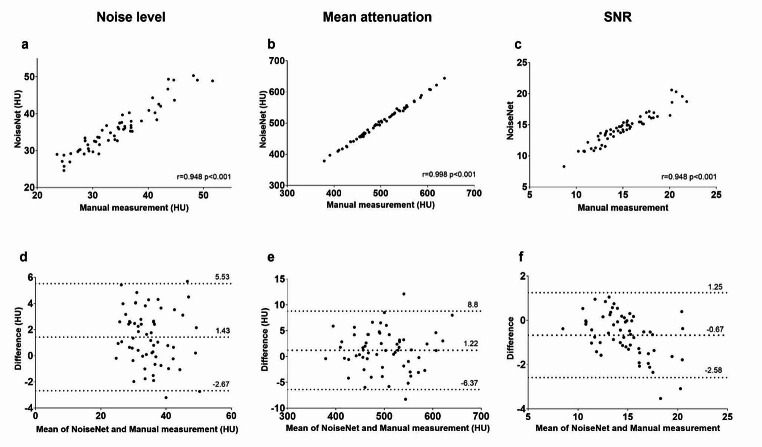
**a-f** Correlation between NoiseNet and manual measurements in mean attenuation (**a**) noise (**b**) and signal-to-noise ratio (SNR, c). The corresponding Bland-Altman plots are shown in (**d**-**f**)

In the test set (*n* = 60), there was a significant positive correlation between NoiseNet and manual measurements considering the three variables attenuation (*r* = 0.998; *p* < 0.001), noise (*r* = 0.948; *p* < 0.001) and signal-to-noise ratio (*r* = 0.948; *p* < 0.001) (Fig. 2a-c). There was a small, systematic and significant difference between NoiseNet and manual measurements, limits of agreement for noise = 1.43 HU (-2.67–5.53; *p* < 0.001), attenuation = 1.22 HU (-6.37–8.80; *p* < 0.05) and signal-to-noise ratio = -0.67 (-2.58– 1.25; *p* < 0.001). The mean absolute difference and mean absolute relative error was 2.04 HU (1.66– 2.43); 6.29% (5.0–7.57) for noise, 3.19 HU (2.55–3.83); 0.64% (0.51–0.76) for attenuation and 0.91 (0.71–1.10); 5.76% (4.71–6.82) for signal-to-noise ratio respectively.

### Influence of noise on image quality

To evaluate the correlation between noise level and image quality, NoiseNet was applied to the previously annotated dataset of 529 CCTA studies. NoiseNet was able to analyze attenuation, noise level and signal-to-noise ratio in 528 out of the 529 cases. One case was excluded because NoiseNet could not detect the ostium of the LM and thereby no analysis was performed. This case was reviewed manually, and we found an anomaly in that specific case where LM originated from the RCA. We assume that this was the reason why NoiseNet could not perform an analysis. Anomalous origin of LM from RCA is a rare condition, in a previous study this anomaly was found in only 0.08% of patients who underwent CCTA [16]. The remaining 528 CCTA studies were distributed in the three image quality subgroups as follows: 158 fully diagnostic; 341 diagnostic with excluded parts and 29 non-diagnostic.

**Fig. 3:**
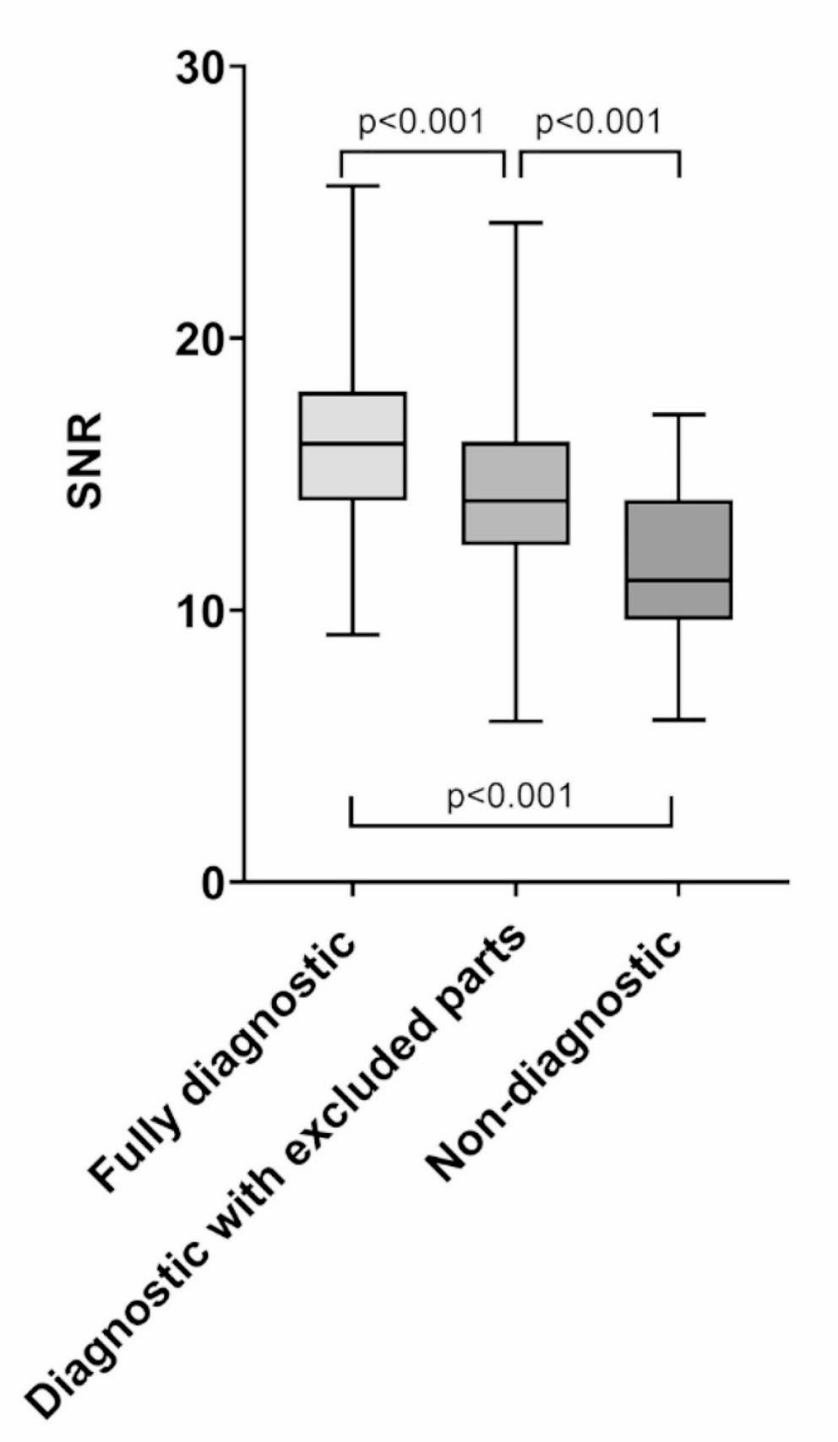
Boxplot showing the signal-to-noise-ratio (SNR) in CCTA studies where diagnostic image quality was judged as fully diagnostic, diagnostic with excluded parts and non-diagnostic

The median noise level in all 528 cases was 35.4 HU (30.9–39.9). The median noise level in the subgroups were: fully diagnostic 33.1 HU (29.8–37.9); diagnostic with excluded parts 36.1 HU (31.5–40.3) and non-diagnostic 42.1 HU (35.2–47.7). Corresponding values for attenuation were: fully diagnostic 533.7 HU (484.7–581.0); diagnostic with excluded parts 502.0 (465.7–552.3) and non-diagnostic 466.0 (421.4–536.3) and for signal-to-noise ratio 16.1 (14.0–18.0); 14.0 (12.4–16.2) and 11.1 (9.6–14.0) respectively (Fig. 3). All differences between subgroups were statistically significant (*p* < 0.001) except the difference in mean attenuation between “diagnostic with excluded parts” and non-diagnostic examinations (*p* = 0.13).

**Fig. 4:**
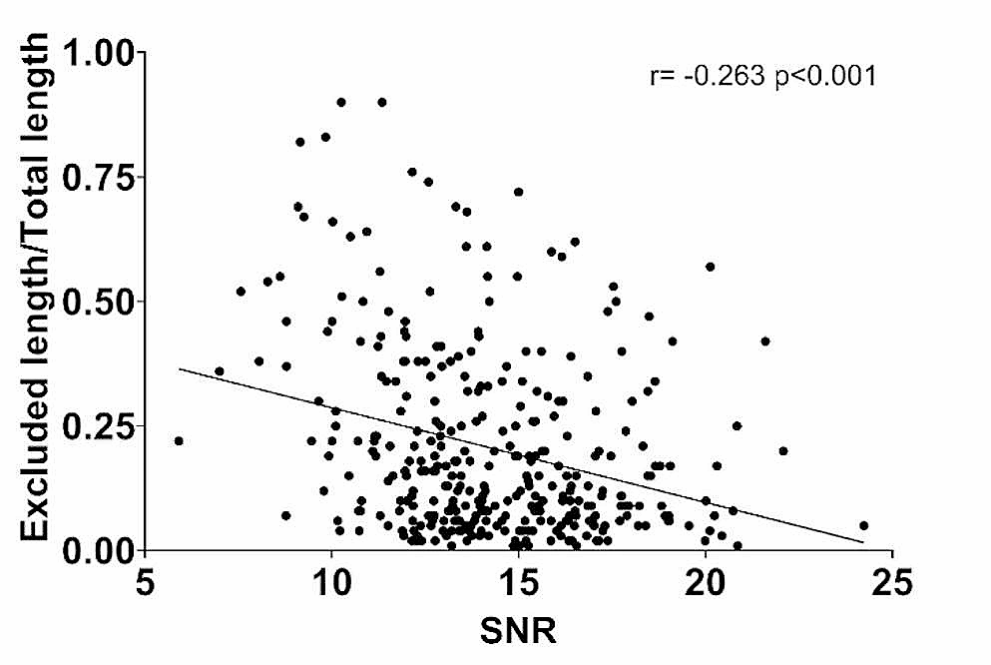
Scatter plot with the subgroup of CCTA studies (*n* = 341) that were diagnostic but had excluded parts due to low image quality. The plot shows the correlation between signal-to-noise ratio (SNR) and the proportion of the coronary artery tree that had been excluded

In the subgroup “diagnostic with excluded parts” (*n* = 341) there was a small but significant positive correlation between noise and excluded vessel length (*r* = 0.264; *p* < 0.001) and a small, significant negative correlation between signal-to-noise ratio and excluded vessel length (*r*= -0.295; *p* < 0.001). When analyzing the correlation between signal-to-noise ratio and the ratio of excluded vessel length and total vessel length, there was a small but significant negative correlation (*r*=-0.263; *p* < 0.001) (Fig. 4).

In 96 cases of 341(28%) of the subgroup diagnostic with excluded parts, the stated reason for exclusion was noise. There was no significant difference in noise level between these 96 cases and the CCTA studies with excluded parts made for other reasons than noise (*n* = 245) (*p* = 0.739).

**Fig. 5:**
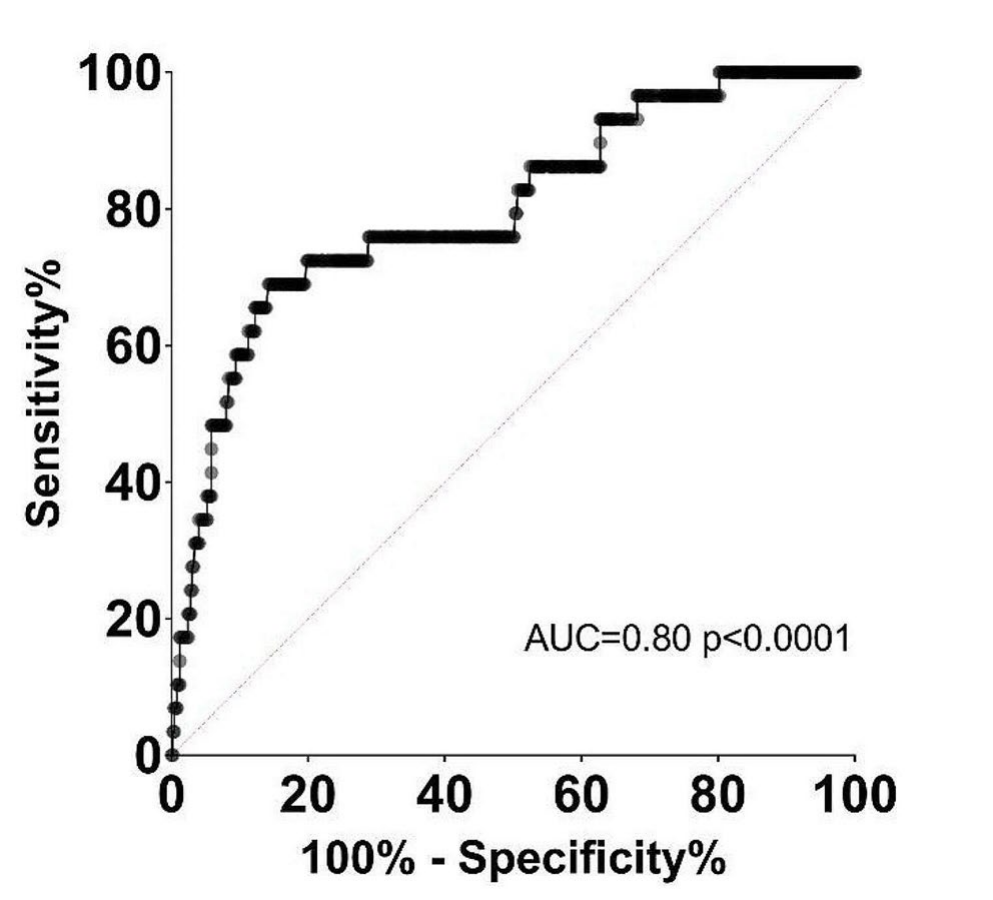
ROC-curve for the performance of signal-to-noise ratio (SNR) in differentiating diagnostic and non-diagnostic examinations. AUC: Area under the curve

ROC analysis of noise level and signal-to-noise ratio for prediction of a non-diagnostic study showed an AUC for noise of 0.73 (CI 0.64–0.83) and AUC for signal-to-noise ratio of 0.80 (CI 0.71–0.89) (Fig. 5). With a proposed cut off value of 38.5 HU for noise, the sensitivity was 65% and the specificity 70% for the detection of a non-diagnostic study. With a proposed cut off value of 12.5 for signal-to-noise ratio, the sensitivity was 72% and the specificity was 81% for detection of a non-diagnostic study.

## Discussion

We developed an automatic CNN-based algorithm that can perform noise measurements in the aortic root on par with a radiologist. Furthermore, we showed that automatic measurements of noise and signal-to-noise ratio can be used to evaluate image quality and find non-diagnostic CCTA studies.

Automatic segmentation of the aortic root has previously been performed to automatically generate anatomical measurements required for transcatheter aortic valve replacement (TAVR) [17] and for measurement of aortic dimensions [18]. In a recent benchmark study, four different CNN architectures for aortic root segmentation were evaluated, yielding a Dice coefficient of 0.954–0.961 [19], which is similar to our algorithm with a Dice coefficient of 0.96. To our knowledge, this is the first study using automatic aortic root segmentation for noise measurements.

The influence of noise on image quality in CCTA has been previously studied. Kim et al. showed that CCTA studies where image quality was visually graded as adequate (moderate image degradation) had significantly higher noise level compared to studies graded as good (minor image degradation) or excellent [4]. The findings are in line with the present study where we found significant differences in noise level between fully diagnostic CCTA studies, diagnostic CCTA studies with excluded parts of the coronary artery tree due to impaired image quality and non-diagnostic CCTA studies with overall poor image quality. Also, there was a significant correlation between length of excluded coronary segments and noise level. The correlation was weak (*r* = 0.264; *p* < 0.001), likely reflecting that many other factors influence image quality, where blurring due to cardiac motion probably is the main reason. Furthermore, image noise was measured at a single point in the aortic root and it is possible that a focal increase in noise in the inferior part of the heart where the liver enters the radiation field might explain some of the excluded segments. Interestingly, there was no significant difference in noise level between subgroups of CCTA studies with exclusions due to noise and exclusions made for other reasons such as motion artifacts. This might reflect that the classification of the reason for non-diagnostic images is difficult to make for the radiologist and is often complex where a combination of several factors influences image quality.

The signal-to-noise ratio was slightly better than noise in predicting image quality with an AUC of 0.80 compared to 0.73. This reflects the fact that signal-to-noise ratio also takes into account the contrast enhancement of the aortic root, which is another factor that influences image quality. With a proposed cut-off value of 12.5 for signal-to-noise ratio, the sensitivity and specificity are 72% and 81% for detecting a non-diagnostic CCTA examination. There are no established cut-offs for sufficient image quality in CCTA but previous authors have suggested a noise level of < 30 HU and attenuation of > 400 HU for a diagnostic examination which corresponds to a signal-to-noise ratio of 13.3 [3] indicating external validity of our proposed cut-off value.

The image quality has been previously shown to influence both the diagnostic value and the performance of automatic analysis of CCTA [2, 20].However, to our knowledge there are no previous studies where automatic measurement of noise has been used. In SCAPIS and other large CCTA research studies, automatic measurements of noise and signal-to-noise ratio may be used to identify the group of examinations with highest noise level/lowest signal-to-noise ratio. In future analyses, this subgroup may be excluded to improve the correlation between CCTA variables and other variables. NoiseNet can be used as a standardized and reproducible method to exclude the examinations with lowest image quality. Furthermore, a future potential use of the algorithm could be in the CT lab during scanning. NoiseNet can automatically perform measurements of noise and signal-to-noise ratio while the patient is still in the scanner and flag scans with low image quality. This may be a signal to the radiographer to consider repeating the scan after modifying the scan parameters.

### Limitations

The study was performed on a research population where all examinations were performed on the same type of scanner and not in a clinical environment. The suggested algorithm and cut-offs have not been validated on other vendors or protocols. For clinical implementation, the model needs to be tested in a clinical setting.

## Conclusions

In conclusion, NoiseNet can perform noise and signal-to-noise ratio measurements with high accuracy. Noise and signal-to-noise ratio impact image quality and automatic measurements may be used in a research setting to identify CCTA studies with low image quality.

## Data Availability

No datasets were generated or analysed during the current study.

## References

[1] Knuuti J, Wijns W, Saraste A, Capodanno D, Barbato E, Funck-Brentano C et al (2020) 2019 ESC Guidelines for the diagnosis and management of chronic coronary syndromes 10.1093/eurheartj/ehz425 31504439

[2] van Diemen PA, Driessen RS, Stuijfzand WJ, Raijmakers PG, Schumacher SP, Bom MJ et al (2020) Impact of scan quality on the diagnostic performance of CCTA, SPECT, and PET for diagnosing myocardial ischemia defined by fractional flow reserve 10.1016/j.jcct.2019.06.007 31302028

[3] Ghekiere O, Salgado R, Buls N, Leiner T, Mancini I, Vanhoenacker P et al (2017) Image quality in coronary CT angiography: challenges and technical solutions 10.1259/bjr.20160567 PMC5605061 28055253

[4] Kim H, Park CH, Han KH, Kim TH (2015) Predicting the image noise level of prospective ECG-triggered coronary computed tomography angiography: quantitative measurement of thoracic component versus body mass index 10.1007/s10554-015-0796-6 26507324

[5] Law WY, Huang GL, Yang CC (2022) Effect of body Mass Index in Coronary CT Angiography Performed on a 256-Slice multi-detector CT scanner. Diagnostics (Basel) 1210.3390/diagnostics12020319 10.3390/diagnostics12020319 PMC8871507 35204410

[6] Benz DC, Benetos G, Rampidis G, von Felten E, Bakula A, Sustar A et al (2020) Validation of deep-learning image reconstruction for coronary computed tomography angiography: impact on noise, image quality and diagnostic accuracy 10.1016/j.jcct.2020.01.002 31974008

[7] Schuhbaeck A, Schaefer M, Marwan M, Gauss S, Muschiol G, Lell M et al (2013) Patient-specific predictors of image noise in coronary CT angiography 10.1016/j.jcct.2012.10.011 23352772

[8] Seppelt D, Kolb C, Kuhn JP, Speiser U, Radosa CG, Hoberuck S et al (2019) Comparison of sequential and high-pitch-spiral coronary CT-angiography: image quality and radiation exposure 10.1007/s10554-019-01568-y 30850908

[9] Bernard A, Comby PO, Lemogne B, Haioun K, Ricolfi F, Chevallier O et al (2021) Deep learning reconstruction versus iterative reconstruction for cardiac CT angiography in a stroke imaging protocol. reduced radiation dose and improved image quality 10.21037/qims-20-626 PMC7719916 33392038

[10] Bergstrom G, Berglund G, Blomberg A, Brandberg J, Engstrom G, Engvall J et al (2015) The Swedish CArdioPulmonary BioImage Study: objectives and design 10.1111/joim.12384 PMC4744991 26096600

[11] Bergstrom G, Persson M, Adiels M, Bjornson E, Bonander C, Ahlstrom H et al (2021) Prevalence of Subclinical Coronary Artery Atherosclerosis in the General Population 10.1161/CIRCULATIONAHA.121.055340 PMC8448414 34543072

[12] Fagman E, Alven J, Westerbergh J, Kitslaar P, Kercsik M, Cederlund K et al (2023) High-quality annotations for deep learning enabled plaque analysis in SCAPIS cardiac computed tomography angiography 10.1016/j.heliyon.2023.e16058 PMC10199173 37215775

[13] Çiçek Ö, Abdulkadir A, Lienkamp SS, Brox T, Ronneberger O (2016). 3D U-Net: learning dense volumetric segmentation from sparse annotation.

[14] Tragardh E, Borrelli P, Kaboteh R, Gillberg T, Ulen J, Enqvist O et al (2020) RECOMIA-a cloud-based platform for artificial intelligence research in nuclear medicine and radiology 10.1186/s40658-020-00316-9 PMC7403290 32754893

[15] Dozat T (2016) Incorporating Neserov Momentum into Adam. Proceedings of the 4th International Conference on learning Representations:1–4

[16] Graidis C, Dimitriadis D, Karasavvidis V, Dimitriadis G, Argyropoulou E, Economou F et al (2015) Prevalence and characteristics of coronary artery anomalies in an adult population undergoing multidetector-row computed tomography for the evaluation of coronary artery disease 10.1186/s12872-015-0098-x PMC4592552 26431696

[17] Toggweiler S, Wyler von Ballmoos MC, Moccetti F, Douverny A, Wolfrum M, Imamoglu Z et al (2024) A fully automated artificial intelligence-driven software for planning of transcatheter aortic valve replacement. Cardiovasc Revasc Med. 10.1016/j.carrev.2024.03.00810.1016/j.carrev.2024.03.008 10.1016/j.carrev.2024.03.008 38467531

[18] Sieren MM, Widmann C, Weiss N, Moltz JH, Link F, Wegner F et al (2022) Automated segmentation and quantification of the healthy and diseased aorta in CT angiographies using a dedicated deep learning approach 10.1007/s00330-021-08130-2 34170365

[19] Yang T, Zhu G, Cai L, Yeo JH, Mao Y, Yang J (2023) A benchmark study of convolutional neural networks in fully automatic segmentation of aortic root 10.3389/fbioe.2023.1171868 PMC10311214 37397959

[20] Xu PP, Li JH, Zhou F, Jiang MD, Zhou CS, Lu MJ et al (2020) The influence of image quality on diagnostic performance of a machine learning-based fractional flow reserve derived from coronary CT angiography 10.1007/s00330-019-06571-4 32006167

